# *Drosophila kikkawai* ortholog of the *D. melanogaster* Muller D element *ash1*

**DOI:** 10.17912/micropub.biology.001322

**Published:** 2024-11-22

**Authors:** Mia Mo, Destiny Sabb, Larissa LoBello, Kayla Chambers, Kacie Kershaw, Cameron Welles, Joshua Kurucz, Caleb Rhyne, Angel Nichols, John Stanga, James E. J. Bedard, Cindy Arrigo

**Affiliations:** 1 Washington University in St. Louis, St. Louis, Missouri, United States; 2 New Jersey City University, Jersey City, New Jersey, United States; 3 Mercer University, Macon, Georgia, United States; 4 St. Joseph’s University, Philadelphia, Pennsylvania, United States; 5 University of the Fraser Valley, Abbotsford, British Columbia, Canada; 6 Biology, Mercer University, Macon, Georgia, United States; 7 Biology, University of the Fraser Valley, Abbotsford, British Columbia, Canada; 8 Biology, New Jersey City University, Jersey City, New Jersey, United States

## Abstract

The *Drosophila kikkawai* feature with Gene ID 108083276 was determined to be an ortholog of *Drosophila melanogaster* absent, small, or homeotic discs 1 ( *ash1* ). Two isoforms, ash1-PB and ash1-PC, were constructed on the *D. kikkawai* Muller D element using the GEP annotation protocol. The second coding exon of *D. kikkawai* *ash1* includes an insertion translated into 18 additional amino acids compared to the *D. melanogaster* protein and is supported by RNA-Seq coverage, the lack of splice junction predictions, and multiple gene predictors. The first intron in both isoforms of *D. kikkawai ash1* contains a well conserved non-canonical GC splice site.

**Figure 1. D. kikkawai feature with Gene ID 108083276 was determined to be an ortholog of D. melanogaster ash1 and possesses a net 18 AA insertion relative to the D. melanogaster ash1 protein:**
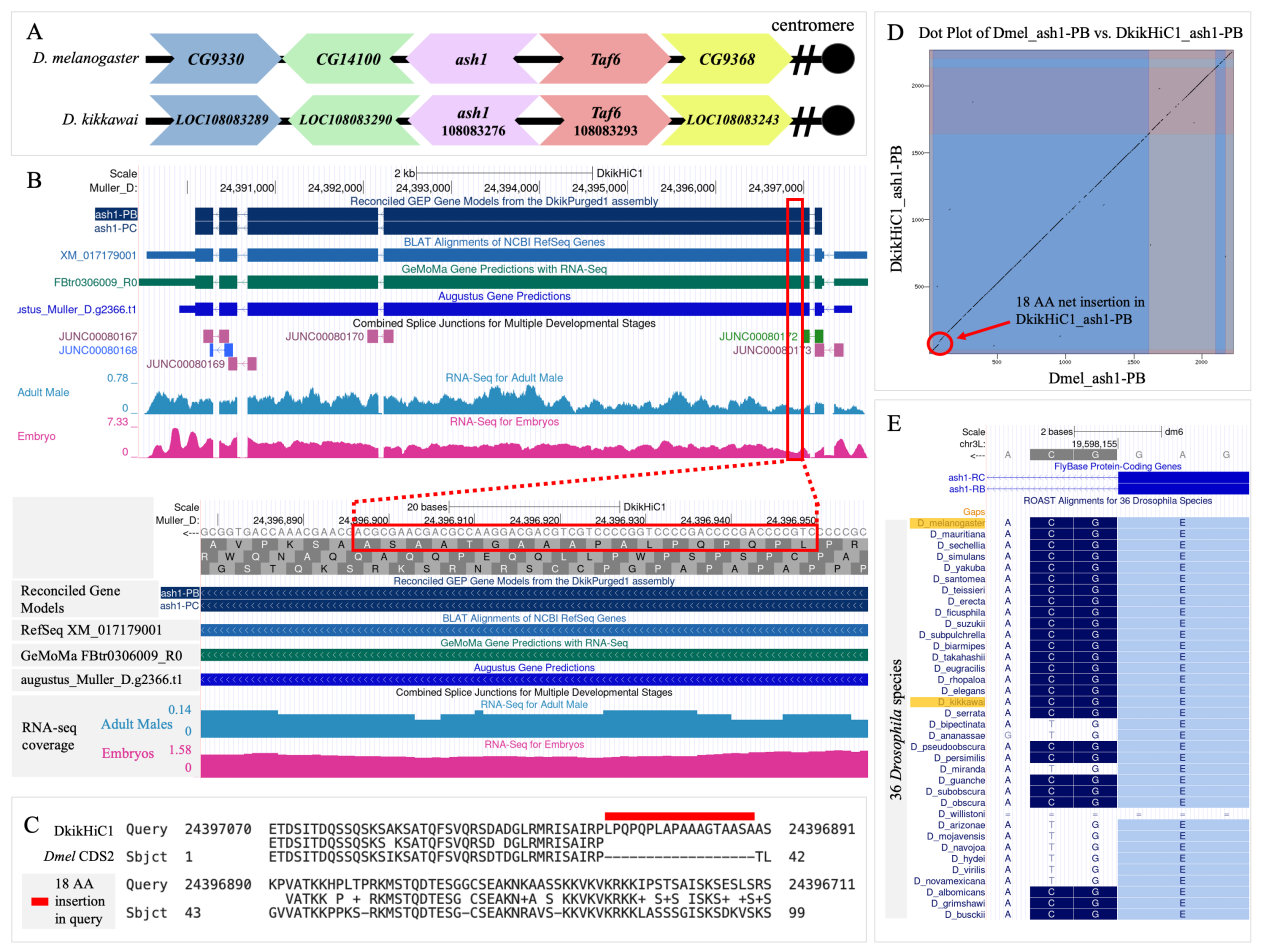
**
(A) Gene neighborhood of *ash1* in *Drosophila melanogaster* (top) and *Drosophila kikkawai* (bottom).** The large chevron arrows are labeled with gene symbols. For *D. melanogaster* , these labels correspond to the FlyBase gene symbols. For *D. kikkawai* , the labels correspond to the gene symbols in the NCBI Gene database. For *D. kikkawai* genes with published gene symbols, the label begins with the gene symbol (e.g., *ash1* ) rather than “LOC”, followed by the NCBI Gene ID (e.g., 108083276). The direction of the chevron arrows corresponds to the orientation of the gene relative to the centromere (black dot) *.* The *ash1* gene is coded on the minus strand in *D. melanogaster* and *D. kikkawai .* Orthologous genes are denoted with the same color chevron arrow. **(B) Genome Browser image of *ash1* gene model(s) on the *D. kikkawai* DkikHiC1 assembly with evidence tracks.** The dark blue gene models at the top of the image represent the final gene models for ash1-PB and ash1-PC, which have identical coding regions. The provided evidence tracks include the BLAT alignment of the *D. kikkawai* RefSeq mRNA XM_017179001 , two gene predictions from GeMoMa and Augustus, mapped splice junctions, and overall RNA-Seq coverage from adult males and embryos. The inset shows the region in the second coding exon (CDS2) of the *D. kikkawai* *ash1* gene (red box) that contains an insertion of 18 amino acids (AA) compared to the *D. melanogaster* CDS2. Note the continuous RNA-Seq coverage, the absence of compatible splice donor and acceptor sites that satisfy the biological constraint for minimum intron size, and the absence of detectable RNA-Seq junction fragments in the combined splice junctions track. **(C)** **BLASTx alignment output comparing translated DNA sequence from CDS2 in *D. kikkawai* (Query; DkikHiC1) to the AA sequence of CDS2 in *D. melanogaster* (Subject; *Dmel* CDS2) *.*** The alignment of the first 99 AA from CDS2 is shown where the match line in the middle denotes similarity between the query and subject input sequences. The dashes in the subject sequence correspond to gaps in the alignment, indicating an insertion in the query sequence. The bold red line above the alignment indicates the predicted 18 AA insertion in CDS2 of the *D. kikkawai* protein relative to the *D. melanogaster* ash1 protein. **(D) Dot plot comparing AA sequences of ash1-PB in *D. melanogaster* (x-axis) and *D. kikkawai* (y-axis).** Black dots in the dot plot correspond to identical residues between the sequences on the x and y axes. Strong similarity is indicated by a continuous black line. The alternating-colored boxes indicate exon boundaries. The red circle demarcates the discontinuity displayed in panels B and C. **(E)** **ROAST alignment of 36 *Drosophila* species shows level of conservation of the non-canonical GC splice donor site for the first intron.** The ROAST alignment depicts conservation of a non-canonical GC donor site across 26 of the 36 *Drosophila* species. The light blue boxes indicate the end of CDS1. The non-canonical GC site is highlighted in dark blue. The first intron of the *D. kikkawai* and *D. melanogaster* *ash1* genes both contain the GC donor site. *D. kikkawai* and *D. melanogaster* are highlighted in yellow.

## Description

The *Drosophila melanogaster* absent, small, or homeotic discs 1 ( *ash1* ) gene (FBgn0005386) is found on chromosome 3L (Muller D element) and encodes a [histone H3]-lysine(36) N-dimethyltransferase (EC 2.1.1.357) which is involved in regulating transcription (Goodliffe et al., 2007) . The *ash1* gene has been assigned to the SET DOMAIN LYSINE METHYLTRANSFERASES (KMT) gene group by FlyBase (FBgg0000274; Jiang et al., 2017; Mohan et al., 2011). The group encodes proteins with a variety of proposed molecular functions including histone methyltransferase activity and chromatin binding (Huang et al., 2017) . Specifically, the ash1 protein enables histone H3K36 methyltransferase activity (GO:0046975), H3K36 dimethyltransferase activity (GO:0140954) and histone H3K4 methyltransferase activity (GO:0042800) (Byrd & Shearn, 2003; Dorighi & Tamkun, 2013; Huang et al., 2017; Tanaka et al., 2007) . The product of *ash1* is a part of the TrxG (Trithorax) protein group. Proteins in this group aid in keeping homeotic genes active by preventing PcG (Polycomb Group) proteins from silencing homeotic genes (Dorafshan et al., 2019) . According to FlyBase (release FB2022_06), *ash1* is orthologous to ASH1 like histone lysine methyltransferase ( *ASH1L* ) in humans with a DIOPT score of 11/14 (Hu et al., 2011; Öztürk-Çolak et al., 2024).

***D. kikkawai* feature with Gene ID 108083276 is the putative ortholog of *ash1* .** The ortholog assignment of *ash1* is supported by local synteny with *D. melanogaster* , protein sequence similarity, and experimental RNA-Seq evidence. *D. melanogaster* *ash1* is surrounded by the genes *CG9330* (FBgn0036888) and *CG14100* (FBgn0036889) downstream, and *Taf6* (FBgn0010417) and *CG9368* (FBgn0036890) upstream. The putative ortholog for *ash1* was determined to correspond to a feature with Gene ID 108083276 on the *D. kikkawai* Muller D element with coordinates that are consistent with the top hit of a tBLASTn (v2.15.0+) search with the *D. melanogaster* ash1-PB protein against the entire *D. kikkawai* DkikHiC1 (GenBank Assembly Accession: GCA_030179895.1 ) assembly (Camacho et al., 2009) . The match with the lowest E-value mapped to scaffold CM058225.1 (assigned to the Muller D element) between *LOC108083290* and *Taf6* (Gene ID: 108083293) with an overall identity of 84.08%, a coverage of 99%, and an E-value of 0.0 (i.e., E-value less than 1x10 ^-180^ ). The second best hit maps to scaffold CM058228.1 (X chromosome) with a substantially lower identity of 40.10%, a coverage of 65%, and a substantially greater E-value of 7x10 ^-36^ . *D. kikkawai ash1* is surrounded by genes with high sequence similarity to the *D. melanogaster* genes *CG9330* (Gene ID: 108083289), *CG14100* (Gene ID: 108083290), *Taf6* (Gene ID: 108083293) and *CG9368* (Gene ID: 108083243) ( Figure 1A ). The consistent local synteny with *D. melanogaster* supports the assignment of the feature with Gene ID 108083276 as *ash1* in *D. kikkawai* . Orthology was further supported by the BLASTp (v2.15.0+) protein alignment between the *D. melanogaster* ash1 protein and the protein product of the final *D. kikkawai* model, which shows a sequence similarity of 89.4% and a percent identity of 83.7%. Nucleotide sequence data reported are available in the Third-Party Annotation Section of the DDBJ/ENA/GenBank databases under the accession numbers TPA: BK068291 and BK068292 .

**Characterizing the net 18 AA insertion in the *D. kikkawai* ash1 protein.** The *ash1* gene in *D. melanogaster* has two isoforms, ash1-PB and ash1-PC, with identical polypeptide sequence. These isoforms have five Coding DNA Sequences (CDS). Comparing the *D. kikkawai* and *D. melanogaster* gene models, both have the same number of CDSs ( Figure 1B ). However, the dot plot of the protein alignment depicts a discontinuity consistent with an insertion in the second CDS of *D. kikkawai* ash1-PB ( Figure 1D ). The cumulative change in this region is an 18 AA insertion relative to the *D. melanogaster* ash1 protein. This extension is one of the contributors to the difference in the total protein length from 2,226 AA in *D. melanogaster* to 2,269 AA in *D. kikkawai* ( Figure 1C ). Due to continuous RNA-Seq coverage across this discontinuity in the alignment, and the lack of compatible splice donor and acceptor sites, the 18 AA difference is not a novel intron. Further analysis would be required to determine if the presence of these new animo acids alters the protein's function in *D. kikkawai* compared to *D. melanogaster .* However, we note that the insertion does not disrupt any of the identified protein domains [AWS domain, SET domain, Bromo adjacent homology (BAH) domain].

**Characterizing the noncanonical GC splice donor site in the first intron of *D. kikkawai ash1* .** In *D. melanogaster* , both the B and C isoforms of *ash1* have a noncanonical GC splice donor site after the end of the first CDS. In *D. kikkawai* , the non-canonical splice site is supported by the substantial decrease in RNA-Seq read coverage and a splice junction prediction derived from spliced RNA-Seq reads (JUNC00080172; score = 79). Based on the ROAST alignment (March 2008 release), this GC donor site appears to be conserved in 26 out of 36 *Drosophila* species that are part of the whole genome multiple sequence alignment, including in *D. kikkawai* ( Figure 1E ). While 10 of the 36 species exhibit the canonical GT donor site (e.g., *D. ananassae , D. bipectinata* , etc.), the conservation of the GC donor site in closely related and more evolutionarily distant *Drosophila* species suggests that the common ancestor of *D. melanogaster* and *D. kikkawai* possessed the non-canonical GC splice donor site.

## Methods

The protocol used to annotate and reconcile the *ash1* gene model and neighboring gene models is described in Rele et al., 2023. The annotations are based on the annotated gene models for FlyBase release FB2022_06 ( *D. melanogaster* release 6.49) in the release 6 assembly (Hoskins et al., 2015) . A mirror of the UCSC Genome Browser (v435) (Kent et al., 2002; Navarro Gonzalez et al., 2021) is maintained by the Genomics Education Partnership (GEP) at https://gander.wustl.edu/. Within the *D. kikkawai* Hi-C genome browser, evidence tracks that display the results of experimental data (e.g., RNA-Seq) and computational tools including tBLASTn, gene predictors, and BLAT were used to support the assignment of the *ash1* ortholog and annotate a gene model. The *D. kikkawai* RNA-Seq data was generated by the modENCODE project (Chen et al., 2014) . The tBLASTn (v2.13.0+) results report the region of the genome with the highest similarity to *D. melanogaster* protein-coding sequences. The NCBI BLAST+ web interface uses v2.15.0+. Gene predictors, such as Augustus (v3.5.0) and GeMoMa (v1.9), provide computational predictions for the structures and locations of putative genes. BLAT (v37x1) alignments report the region of the genome with the highest similarity to *D. melanogaster* transcripts.

## Extended Data

Description: Transcript, peptide, and generic feature format version 3 (GFF3) files for all isoforms (B and C) of ash1 for DkikHiC1 assembly. Resource Type: Dataset. DOI: 10.22002/sdh1e-5wn19
